# Low-Temperature Plasma-Assisted Nitrogen Fixation for Corn Plant Growth and Development

**DOI:** 10.3390/ijms22105360

**Published:** 2021-05-19

**Authors:** Pradeep Lamichhane, Mayura Veerana, Jun Sup Lim, Sohail Mumtaz, Bhanu Shrestha, Nagendra Kumar Kaushik, Gyungsoon Park, Eun Ha Choi

**Affiliations:** 1 Plasma Bio-Science Research Center, Applied Plasma Medicine Center, Department of Electrical and Biological Physics, Kwangwoon University, Seoul 01897, Korea; theprodip@gmail.com (P.L.); mayuraveerana@gmail.com (M.V.); junsup117@nate.com (J.S.L.); sohail.ahmed2015@gmail.com (S.M.); kaushik.nagendra@kw.ac.kr (N.K.K.); gyungp@kw.ac.kr (G.P.); 2 Department of Electronic Engineering, Kwangwoon University, Seoul 01897, Korea; bnu@kw.ac.kr

**Keywords:** plasma-assisted nitrogen fixation, chlorophyll, proteins, non-thermal atmospheric pressure nitrogen plasma, plasma-activated water, germination rate, seedling growth

## Abstract

Nitrogen fixation is crucial for plants as it is utilized for the biosynthesis of almost all biomolecules. Most of our atmosphere consists of nitrogen, but plants cannot straightforwardly assimilate this from the air, and natural nitrogen fixation is inadequate to meet the extreme necessities of global nutrition. In this study, nitrogen fixation in water was achieved by an AC-driven non-thermal atmospheric pressure nitrogen plasma jet. In addition, Mg, Al, or Zn was immersed in the water, which neutralized the plasma-treated water and increased the rate of nitrogen reduction to ammonia due to the additional hydrogen generated by the reaction between the plasma-generated acid and metal. The effect of the plasma-activated water, with and without metal ions, on germination and growth in corn plants (*Zea Mays*) was investigated. The germination rate was found to be higher with plasma-treated water and more efficient in the presence of metal ions. Stem lengths and germination rates were significantly increased with respect to those produced by DI water irrigation. The plants responded to the abundance of nitrogen by producing intensely green leaves because of their increased chlorophyll and protein contents. Based on this report, non-thermal plasma reactors could be used to substantially enhance seed germination and seedling growth.

## 1. Introduction

A recent United Nations Food and Agriculture Organization (FAO) survey reported that world grain production is approximately 2.216 billion tons, while demand hit 2.254 billion tons, and 9 million people continue to suffer from hunger [1]. Sooner or later, global food shortages will increase because the amount of land available for cultivation is continuously being reduced by infrastructure, industrialization, and urbanization. To fulfill the demand for food, crop yields should be increased via an economic, sustainable, and feasible process. It is obvious that increasing seed germination rates and plant growth will increase crop yields [2]. The main reason for low crop yields is often connected to fertilizer limitations [3]. Nitrogen availability often limits crop production [4]. Adding fixed nitrogen to crops in the form of fertilizers enhances food production, and thus researchers are actively pursuing artificial nitrogen fixation mechanisms [5].

The nitrogen fixation process is initiated by the disassociation of the nitrogen molecule (N ≡ N) [6]. The nitrogen triple bond is one of the strongest bonds because of its vibrational excitation energy being 1.5–4 eV (1.4 × 10${}^{5}$–3.8 × 10${}^{5}$ J/mol) [6], bond dissociation energy approximately 9.1 eV $(8.7\times 10^{5}$ J/mol) [7], and ionization energy approximately 14.53 eV $(1.39\times 10^{6}$ J/mol) [8,9]. Sufficient energy must be supplied to break the bond for artificial nitrogen fixation. Industrial nitrogen fixation was first carried out approximately a century ago by passing air through an electric arc to create nitric acid (Birkeland–Eyde process) [10]. This method was not sustained for very long because of the high demand for energy (2–5 MJ/mol) and the inaccessibility of renewable energy sources at that time [11]. Currently, the Haber–Bosch (*H*–*B*) process is used, but although this consumes only approximately 0.5–0.9 MJ/mol of energy, some environmental, establishment, production, and transportation cost-related issues remain [11,12,13]. Nowadays, non-thermal atmospheric pressure plasma (NTAPP) technology is considered a possible replacement for conventional nitrogen fixation because it fixes atmospheric nitrogen in the form of nitric oxide (NO), nitrite (NO${}_{2}$), nitrate (NO${}_{3}$), di-nitrogen trioxide (N${}_{2}$O${}_{3}$), dinitrogen pentoxide (N${}_{2}$O${}_{5}$), and ammonia (NH${}_{3}$) [14,15,16,17], which can all be used as fertilizers [18]. The theoretical energy consumption rate of nitrogen fixation using a NTAPP is the lowest among all the existing artificial and natural processes nitrogen fixation, being only approximately 0.2 MJ/mol [11]. These nitrogen derivatives are the mobile and soluble forms of nitrogen in soil, which makes them more suitable for plant uptake [19,20]. NTAPP methods could prove to be superior to existing fertilizer technology for the following reasons: (i) They operate under atmospheric conditions using sustainable energy [21,22]. (ii) In contrast to the H-B process, they do not emit any greenhouse gases [23,24]. (iii) They are cost effective in terms of establishment, production, carriage, and storage [7]. (iv) Finally, localized production (on-site on-demand), for example, on farmland, is also feasible [9].

The literature on plasma agriculture details the effectiveness of various approaches for NTAPP, from seed to field [25,26,27]. In general, water treated by exposure to a plasma discharge significantly affects seed germination and seedling growth [28,29,30,31,32]. Plants irrigated with plasma-treated water also generate increased crop yields [33,34,35]. These literature reports claim that plasma-treated water activates the synthesis of plant hormones such as auxin and cytokinin, inducing other physicochemical changes to enhance the germination, growth, and development of plants. Separately, a significant amount of research has also been already conducted on the development and improvement of plasma-assisted nitrogen fixation [36,37,38,39,40]. Nitrogen-fixed water is acidic (∼pH 2–3) because of the dissolved nitrogen oxides and hydrogen peroxide [41]. Direct soaking of plants in such highly acidic plasma-treated water could be harmful to crop yields. Therefore, it is necessary to develop an alternative method that can produce nitrogen-rich water under neutral conditions.

**Figure 1:**
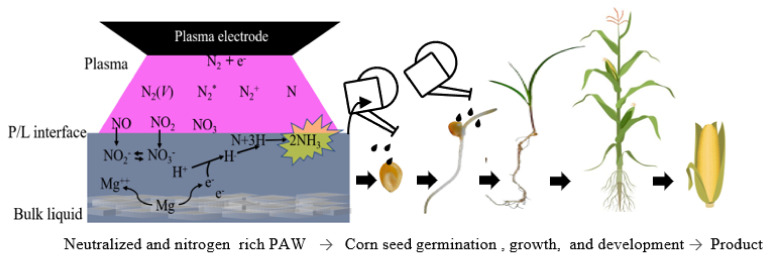
Schematic representation of the experiment design.

In this study, air, metal, and water are used to produce a liquid fertilizer using a non-thermal plasma, a system that has excellent potential for coupling with on-site cultivation systems for on-demand use. The process can be coupled to soil-based or hydroponic cultivation systems. Some of the metals that are essential for plants—magnesium (Mg), zinc (Zn), and aluminum (Al)—were individually dissolved in the plasma-treated water to control its acidity. It was found that these metals not only controlled the acidity of the plasma-treated water, but also enhanced the nitrogen fixation rate. A schematic representation of the experiment design is shown in Figure 1. In the current work, the plasma-activated water with and without (PAW w/wo) metal were used to irrigate corn (*Zea mays*), and the effects of this water on seed germination and plant growth were examined. Subsequently, the chlorophyll, plant protein, nitrate–nitrogen, and ammonium ion contents of the plant tissue were also determined.

## 2. Results

### 2.1. Electrical and Optical Properties of the Plasma Jet

**Figure 2:**
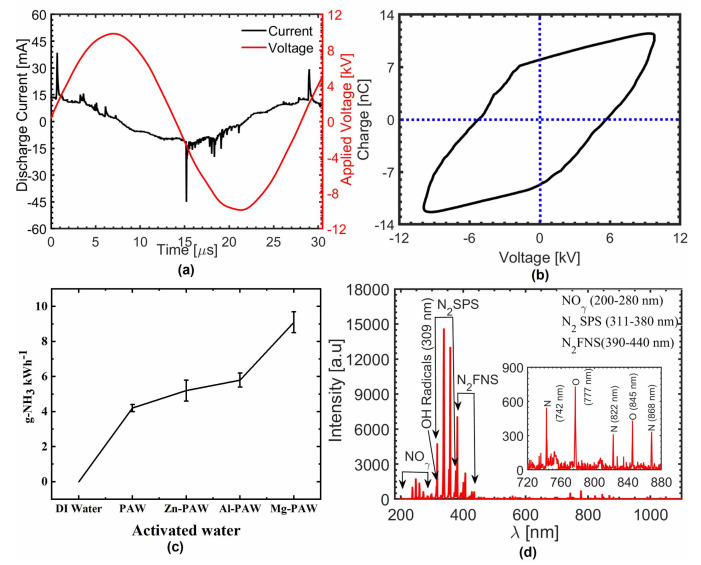
(**a**) Typical current and voltage waveforms of the plasma discharge. (**b**) Corresponding Q–V Lissajous figure. (**c**) The energy consumption for ammonia synthesis with various metal immersed in water. (**d**) Optical emission spectroscopy (OES) results for the nitrogen plasma measured at a position 3 mm downstream of the nozzle of the quartz tube. Applied voltage, 10 kV; Frequency, 33 kHz; Working gas, nitrogen.

The typical current and voltage waveforms of our AC-driven plasma system are shown in Figure 2a. The discharge current peaks appear at the positive half cycle of the applied voltage V, which is caused by the nonuniformity of dielectric barrier thickness; subsequently, the current trend is reversed in negative half cycle, with the enhanced electric field due to the accumulation of wall charge(Q) [42,43]. The corresponding Q versus V plot (Lissajous curve) of the current–voltage waveform is shown in the Figure 2b. The total charge transferred over one full period is equal to zero; thus, the Lissajous curve appears as a closed loop resembling a parallelogram [44]. The area within the loop in this plot is the energy dissipated (E) in each cycle [45,46], which was computed to be approximately 220 J in this experiment. The frequency (f) of power source was 33 kHz, and thus 7.2 watt power (= E×f) was dissipated during these experiments.

Plasma-activated water (PAW) was prepared with and without various metals in this experiment. The plasma-activated water without any metal was termed PAW, while those waters prepared with immersed Zn, Al, and Mg in plasma-treated water were named Zn-PAW, Al-PAW, and Mg-PAW, respectively. The same plasma source was used for all the experiment conditions, and the plasma temperature and electron density were measured to be approximately 1.16 eV and $5.23\times 10^{13}$ cm${}^{-3}$, respectively, at the nozzle of the tube; this was estimated by convective wave packet model [47,48]. The distance between plasma/liquid interface and upper surface of the immersed metal was more than 3 cm in the water container. Due to the sufficient separation, no significant changes in electrical properties of plasma has been seen after inclusion of metal in the water. Energy required for plasma-assisted nitrogen reduction to ammonia with various metal in water is plotted in Figure 2c. The highest energy yield of 9 g −NH${}_{3}$ kWh${}^{-1}$ was found with Mg. Under similar conditions, and without any metal, it was approximately 4 g −NH${}_{3}$ kWh${}^{-1}$.

The optical emission spectroscopy (OES) of the plasma source is shown in Figure 2d. The plasma includes OH radicals, which produce OES peaks at 306–310 nm due to the $A^{2}\sum^{+}\left(v^{\prime \prime }=0\right)$−$X^{2}\prod \left(v^{\prime \prime }=0\right)$] transition [49,50]. In addition, intense OES peaks are assigned to the nitrogen second positive system (N${}_{2}$ SPS) at 311–380 nm, which corresponds to the N${}_{2}$(C${}^{3}\prod_{g}-B^{3}\prod_{g}$) transition [49,51,52], and the first negative system of nitrogen (N${}_{2}$ FNS) at 380–420 nm, which corresponds to the N${}_{2}^{+}\left(B^{2}\sum_{u}^{+}-X^{2}\sum_{u}^{+}\right)$ transition [53,54]. Weak emission signals produced by NO${}_{\gamma }$ (200–280 nm), atomic oxygen (777 and 845 nm), and atomic nitrogen (742, 822, and 868 nm) are also apparent in the insects of Figure 2d.

### 2.2. Measurement of Chemical Properties of Plasma-Treated Water

Non-thermal plasmas are emerging as feasible options for producing a variety of reactive oxygen and nitrogen species (RONS) in the liquid phase. Plasma-treated water contains a variety of RONS that play an important role in physiological activation. The DI water was treated using a NTAPP for 10 min and then used to irrigate corn seed, for germination, and the plant seedlings. After the plasma treatment, the temperature of the water was found to be increased by 2–4 ${}^{\circ }$C. The major chemical elements present in the water and the pH values are listed in Table 1. This table gives the NO_X_, hydrogen peroxide (H${}_{2}$O${}_{2}$), and ammonia (NH${}_{3}$) concentrations present of the DI water, PAW, Zn-PAW, Al-PAW, and Mg-PAW samples. The NH${}_{3}$ concentration and pH are higher in Mg-PAW than in the other PAW w/wo metals and DI water. Acidity tends to neutralize in presence of metal in plasma-treated water.

### 2.3. Effect of Irrigation With Various Activated Waters on Seed Germination and Plant Growth

**Figure 3:**
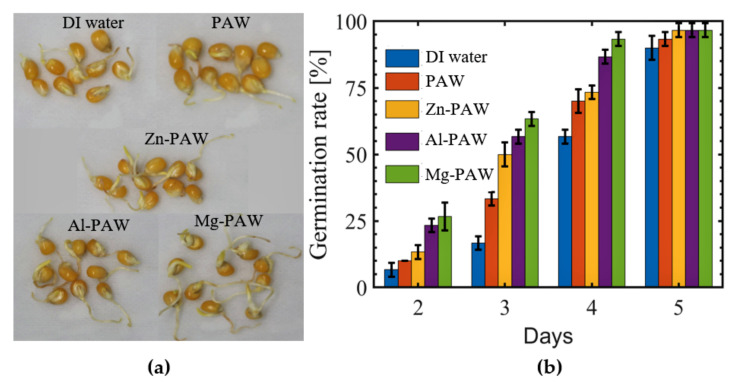
Germination of corn seeds watered with DI water, PAW, PAW with Zn (Zn-PAW), PAW with Al (Al-PAW), or PAW with Mg (Mg-PAW) after sowing on cotton. (**a**) Photograph of Seedling growth on the 4th day after sowing. (**b**) Average germination rate at the end of the 2nd, 3rd, 4th, and 5th days after sowing.

The effect of irrigation using PAW w/wo metals on the germination rate and growth of corn seeds was investigated, and the obtained results were compared with those produced by DI water as the control. The seeds were considered to have germinated when a radical of more than 2 mm in length had been observed. Figure 3a shows a photograph depicting the results of watering corn seeds with DI water, PAW, Zn-PAW, Al-PAW, or Mg-PAW throughout the 5 days after sowing on wet cotton. The germination rate for each day is plotted in Figure 3b. The seeds treated with PAW w/wo metals had significantly enhanced germination rates, but the use of the Mg-PAW and Zn-PAW resulted in more than 90% germination in 4 days, whereas the Al-PAW- and PAW-treated samples required 5 days to exceed the 90% germination rate. Different reactive species and radicals present in the PAW penetrate the seed coat and affect the metabolic processes underlying plant growth [2]. In addition, the activated waters also triggered the growth of the plants by providing different chemical species. RONS triggered the formation of gibberellic acid (GA), which encourages seed germination by improving the growth potential of the germinating seeds and breaks the physico-chemical barriers of the seed coat [55].

**Figure 4:**
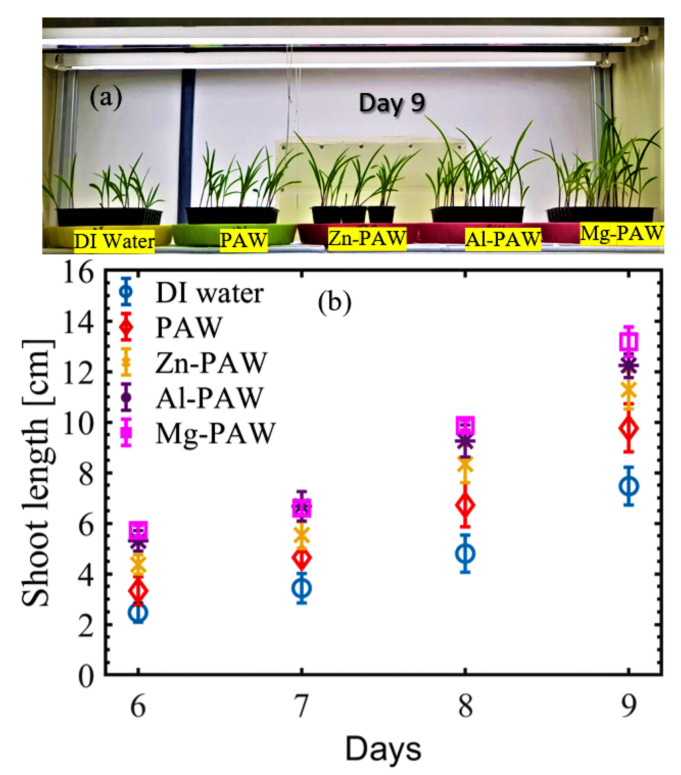
The effect of watering corn plants with DI water, PAW, PAW with Zn (Zn-PAW), PAW with Al (Al-PAW), and PAW with Mg (Mg-PAW). (**a**) Photograph of sample plants watered with various activated waters and the control treatment (DI water) depicting growth on the 9th day. (**b**) Average stem length (including leaf) on days 6, 7, 8, and 9.

To understand the growth and development of the plant after germination, 10 mL of the same water was used to irrigate each corn plant every 48 h. The growth of each plant was recorded each day after the 6th day after sowing in vermiculite. Figure 4a shows the photograph of the planted corn in pots on the 9th day after cultivation, and in Figure 4b, the average measured shoot length (including the leaf) at each day is plotted for all the experimental conditions. In each case, the shoot length increased gradually with the number of days, but the growth was higher in the plants watered with PAW w/wo metal, as compared to those watered with DI water. The PAW with metal treatments resulted in higher growth rates than the PAW without metal treatment; among metal, the maximum growth was generated by the Mg-PAW-treated plants, and the minimum growth rate was observed for the Zn-PAW-treated plants. Shoot lengths on the 9th day were found to be approximately 6 cm on average with DI water, but with PAW, Zn-PAW, Al-PAW, or Mg-PAW these was found to be approximately 7.3, 10.2, 12.3, or 13.2 cm, respectively, on average.

**Figure 5:**
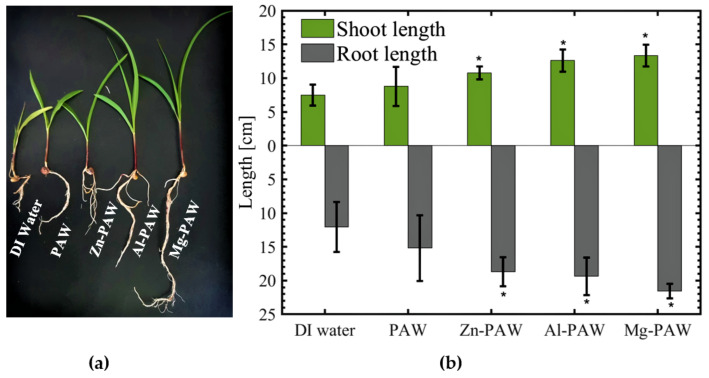
(**a**) Photograph of corn plants and (**b**) average shoot (including leaf) and root lengths on the 10th day after sowing on vermiculite, and after soaking in DI water, only PAW, PAW with Zn (Zn-PAW), PAW with Al (Al-PAW), or PAW with Mg (Mg-PAW). The symbol * is for t-test result which rejects the null hypothesis at the 5% significance level.

On the 10th day, the plant was carefully extracted from the vermiculite. The photograph of complete plant with its root intact on the 10th day is shown in Figure 5a, and the corresponding measurements of the root and shoot lengths are plotted in Figure 5b. The average shoot length was increased by 17%, 43%, 68%, and 78% by treatment with PAW, Zn-PAW, Al-PAW, and Mg-PAW, respectively, as compared to the DI water treatment control condition. As for the shoot length, the average root length with DI water is approximately 10.75 cm, but with the PAW, Zn-PAW, Al-PAW, and Mg-PAW treatments, the root length was increased to 14.3, 17.2, 18.4, and 20.5 cm, respectively.

**Figure 6:**
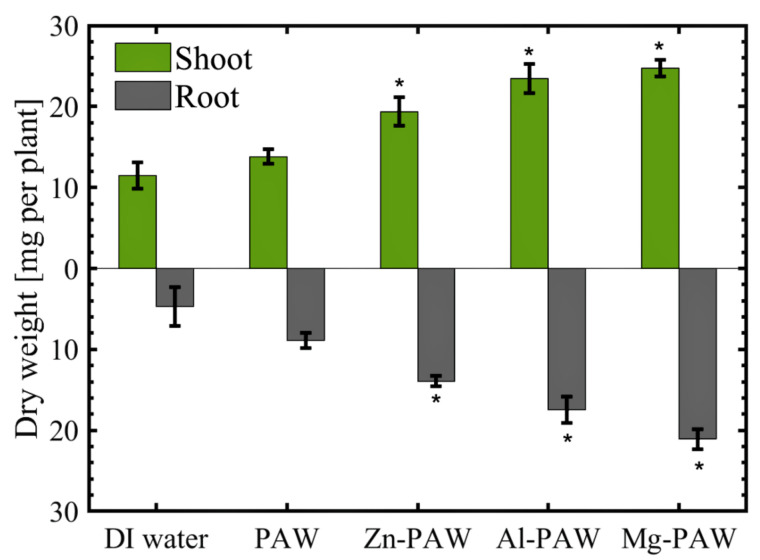
Average dry weight of shoot and roots after the 10th day of soaking in DI water, PAW, PAW with Zn (Zn-PAW), PAW with Al (Al-PAW), or PAW with Mg (Mg-PAW). The symbol * is for t-test result above the 5% significance level.

Most of an organism’s weight is contributed by water in all fresh plants, and the exact amount of water in plants differs according to the quantity of water absorbed. Measurement of plant growth via the dry weight is more reliable than measurement via the fresh weight, due to the presence of water. The variation in dry weight with the use of various types of water is shown in Figure 6. The trends were exactly similar with root and shoot length. In this work, the maximum dry weight of the plant shoots was found to be generated using Mg-PAW (24.7 mg/plant), and with DI water this value was found to be 11.5 mg/plant. Lengthier and more branched roots were observed for the plants irrigated with PAW w/wo metal, with respect to those seen for the plants irrigated with DI water. The dry weight of the root was 4.7, 8.9, 13.6, 17.4, and 21 mg per plant for the DI water, PAW, Zn-PAW, Al-PAW, and Mg-PAW treatments, respectively. The longest roots and shoots were observed with the Mg-PAW irrigation. Furthermore, the leaves were the most greenest with the Mg-PAW irrigation, among the other treatments. Based on this experiment, it can be reasonably suggested that the neutralized PAWs containing metal ions, obtained by metal immersion, not only have a positive impact on seed germination also enhance growth rates and the development of the plant.

### 2.4. Measurement of Chemical Changes in Plant Tissue

**Figure 7:**
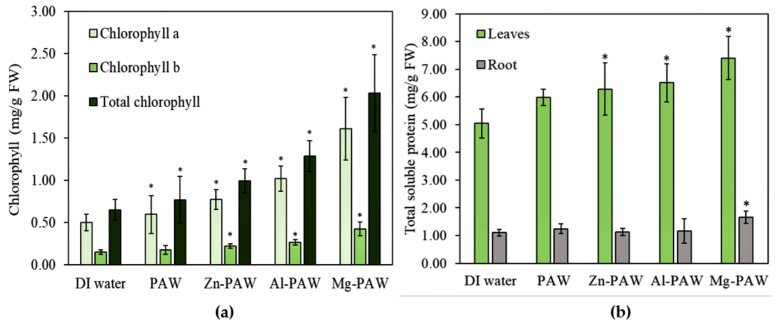
(**a**) Chlorophyll content in leaves and (**b**) total soluble protein content in leaves and roots of fresh corn plants after the 10th day of soaking in DI water, PAW, PAW with Zn (Zn-PAW), PAW with Al (Al-PAW), or PAW with Mg (Mg-PAW). The symbol * represents a t-test result with a significance level greater than 5%.

The chlorophyll content in fresh leaves and the soluble protein present in both the roots and leaves of the corn plants after the 10th day of soaking in DI water or PAW w/wo metal are reported in Figure 7. Trends of increasing chlorophyll (chlorophyll a, chlorophyll b, and total chlorophyll) and protein content with growth rate were seen with DI water, PAW, Zn-PAW, Al-PAW, and Mg-PAW. In addition, the corn seedlings irrigated using PAWs with metals are deep green in color, as compared to the DI water, which is due to the increase in total chlorophyll. Both chlorophyll a and chlorophyll b, and the protein in the leaf are lowest for the DI water treatment and highest for the Mg-PAW treatment. Compared to the effect in the leaves, the total soluble protein content in the root is not significantly enhanced by the use of PAW w/wo metals. In Figure 7a it can be seen that with the DI water treatment, the chlorophyll a and chlorophyll b content were only approximately 0.5 and 0.15 mg/g, respectively, in the fresh leaves, but with the Mg-PAW treatment, these values increased to 1.6 and 0.42 mg/g, respectively.

The nitrogen content of the plants irrigated with PAW w/wo metals are assimilated by the plant for chlorophyll generation and protein synthesis. Figure 7b reflects the total soluble protein contained in the 1 g of fresh plant. Total soluble protein in leaf was only 5 mg with DI water but with PAW, Zn-PAW, Al-PAW, or Mg-PAW, it was increased by 18%, 24%, 29%, or 46%, respectively. However, its concentration in root with DI water was approximately 1 mg which is almost similar with PAW, Zn-PAW, and Al-PAW but with Mg-PAW it was 1.66 mg. Nitrogen supplementation had a substantial effect on the growth and development of the plants. Plants with higher protein synthesis rates are expected to produce greater crop yields. Nitrogen is also a fundamental constituent of chlorophyll, which is involved in the photosynthetic apparatus, as well as carboxylation enzymes and protein membranes [56]. Rates of photosynthesis are directly proportional to chlorophyll content; thus, increased chlorophyll has a positive effect on protein synthesis [57].

**Figure 8:**
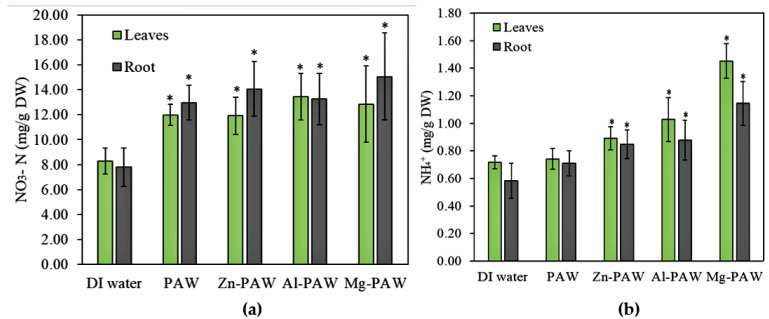
(**a**) NO${}_{3}$-N (**b**) NH${}_{3}$ or NH${}_{4}^{+}$ present in the root and shoot tissues of dry corn plants after the 10th day of soaking in DI water, PAW, PAW with Zn (Zn-PAW), PAW with Al (Al-PAW), or PAW with Mg (Mg-PAW). The symbol * is for t-test result above the 5% significance level.

In the subsequent experiment, NO${}_{3}$-N (nitrate–nitrogen) and the NH${}_{3}$ or NH${}_{4}^{+}$ (ammonia or ammonium ion) present in the dry plant tissues were also measured, and these are presented in Figure 8. Nitrogen derivatives in root and shoots are comparable to each other in all trials. The effect of the metal ion on NO${}_{3}$-N uptake by each plant is not seen clearly from the Figure 8a. Their concentrations were similar to all PAW w/wo metal, which was significantly higher than control. With DI water, NO${}_{3}$-N concentration in 1 g of dry weight was in the range of 8 to 12 mg but with PAW w/wo metal its range was approximately 12 to 18 mg. The NH${}_{3}$ or NH${}_{4}^{+}$ concentrations present in both tissues were found to be increased in the plants treated with PAW w/wo metal as compared to those treated with DI water. Which is shown in Figure 8b. Increasing trend of NH${}_{3}$ or NH${}_{4}^{+}$ in the plant tissue are in accordance with its concentration in irrigated water. The NH${}_{3}$ or NH${}_{4}^{+}$ concentrations both in roots and leaves of plant which are irrigated by Mg-PAW are more than 2 times higher than control sample. Based on this result, addition of metal in plasma treated water significantly enhance the nitrogen content in the plant tissue.

The inclusion of metals in the PAW can significantly affect the physio-chemical properties of the seedling. The NH${}_{3}$ or NH${}_{4}^{+}$ and NO${}_{3}$-N concentrations produced by the Mg-PAW treatment were the greatest among those produced by all the treatments; however, these values are relatively low in DI water and only PAW-irrigated plant. A moderate and similar effect on these was seen for the treatments with Zn-PAW and Al-PAW. These absorbed nitrogen-based products are utilized for chlorophyll and protein syntheses, as well as for nucleic acid synthesis. Thus, the growth rate and germination rate increased after irrigation with PAW w/wo metals.

## 3. Discussion

The enhancement of plasma-assisted nitrogen fixation by nascent hydrogen produced by the immersion of metals in plasma-treated water has been already discussed in detail in our previous report [7]. From agricultural perspective, there are three benefits for the use of metals in plasma-treated water: (i) The metal removes H${}^{+}$ ions, converting them into nascent hydrogen, which modulates the acidity of the plasma treated water. (ii) Newly reduced (nascent) hydrogen is a strong reducing agent, and thus the reduction of nitrogen to ammonia will be increased. (iii) Metal ions present in water results in positive physiological changes in the plant. The mechanisms underlying these processes are described in this section.

In the plasma, the N ≡ N molecule is excited [16] or dissociated [58] by electron impact collisions. Further excited nitrogen molecules can be dissociated upon delivery of additional energy by the plasma [59]. These processes are shown in Equations (1)–(3).

(1) $$N_{2}+e\to 2N+e.$$

(2) $$\begin{array}{c} N_{2}+e\to N_{2}^{*}+e. \end{array}$$

(3) $$\begin{array}{c} N_{2}^{*}+e\to 2N+e. \end{array}$$

**Figure 9:**
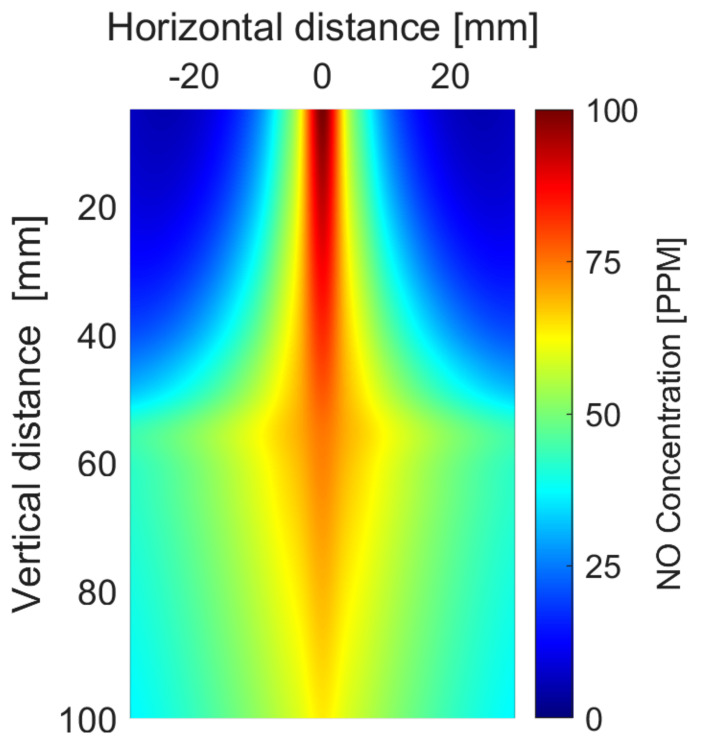
Spatial distribution of NO below the nozzle of the quartz tube.

Atomic nitrogen is further oxidized into NO in presence of OH radicals [60]. NO is the dominant gaseous species generated by the nitrogen plasma jet. The lifetime of NO is approximately 1.2 s, which is limited by presence of other radicals [22]. NO distribution in plasma is shown in Figure 9. These NO densities were recorded in absence of water and metal. In the figure, horizontal 0 is the position of the plasma plume and vertical 0 is for the level of the nozzle of the quartz tube. At the nozzle of the quartz tube, NO density was more than 100 ppm but with horizontal and vertical distance, the density decreases continuously due to the diffusion of atmospheric pressure and further oxidation. It is oxidized by OH radicals to form NO${}_{2}$ and NO${}_{3}$ (Equations (4)–(6)) [5,7]. NO_X_ has a central role in several plant physiological functions [61].

(4) $$\begin{array}{c} N+OH\to NO+H. \end{array}$$

(5) $$\begin{array}{c} NO+OH\to NO_{2}+H. \end{array}$$

(6) $$\begin{array}{c} NO_{2}+OH\to NO_{3}+H. \end{array}$$

NO_X_ is instantly liquefied, giving rise to the formation of NO${}_{2}^{-}$, NO${}_{3}^{-}$, and H${}^{+}$. Typically, H${}^{+}$ is formed via the mechanism outlined in Equations (7)–(10), and this renders plasma-treated water acidic [41,62,63].

(7) $$\begin{array}{c} NO+OH\to NO_{2}^{-}+H^{+}. \end{array}$$

(8) $$\begin{array}{c} NO_{2}+OH\to NO_{3}^{-}+H^{+}. \end{array}$$

(9) $$\begin{array}{c} 2NO_{2}+H_{2}O\to NO_{2}^{-}+NO_{3}^{-}+2H^{+}. \end{array}$$

(10) $$\begin{array}{c} NO+NO_{2}+H_{2}O\to 2NO_{2}^{-}+2H^{+}. \end{array}$$

The metals dipped in water are also oxidized, as outlined in Equations (11)–(13) [64,65]:

(11) $$\begin{array}{c} Zn⇌Zn^{++}+2e^{-}. \end{array}$$

(12) $$\begin{array}{c} Al⇌Al^{+++}+2e^{-}. \end{array}$$

(13) $$\begin{array}{c} Mg⇌Mg^{++}+2e^{-}. \end{array}$$

The metal ions convert NO${}_{2}^{-}$ and NO${}_{3}^{-}$ into metal nitrates and nitrites (Equations (14)–(19)) [64,65]. Metal nitrates and nitrites are neutral salts.

(14) $$\begin{array}{c} Zn^{++}+NO_{2}^{-}⇌Zn{\left(NO_{2}\right)}_{2}. \end{array}$$

(15) $$\begin{array}{c} Zn^{++}+NO_{3}^{-}⇌Zn{\left(NO_{3}\right)}_{2}. \end{array}$$

(16) $$\begin{array}{c} Al^{+++}+NO_{2}^{-}⇌Al{\left(NO_{2}\right)}_{3}. \end{array}$$

(17) $$\begin{array}{c} Al^{+++}+NO_{3}^{-}⇌Al{\left(NO_{3}\right)}_{3}. \end{array}$$

(18) $$\begin{array}{c} Mg^{++}+NO_{2}^{-}⇌Mg{\left(NO_{2}\right)}_{2}. \end{array}$$

(19) $$\begin{array}{c} Mg^{++}+NO_{3}^{-}⇌Mg{\left(NO_{3}\right)}_{2}. \end{array}$$

Electrons from the metal reduce H${}^{+}$ to H, as in Equation (20) [23]. Presence of metal in plasma-treated water significantly increase the pH value (= −log[H${}^{+}$]) due to the reduced H${}^{+}$.

(20) $$\begin{array}{c} H^{+}+e^{-}\to H↑. \end{array}$$

When H is encountered by N, N is reduced to NH${}_{3}$ via the process shown in Equations (21) and (22) [16,41,66,67]:

(21) $$\begin{array}{c} N+3H\to NH_{3}. \end{array}$$

(22) $$\begin{array}{c} N_{2}^{*}+3H_{2}\to 2NH_{3}. \end{array}$$

**Figure 10:**
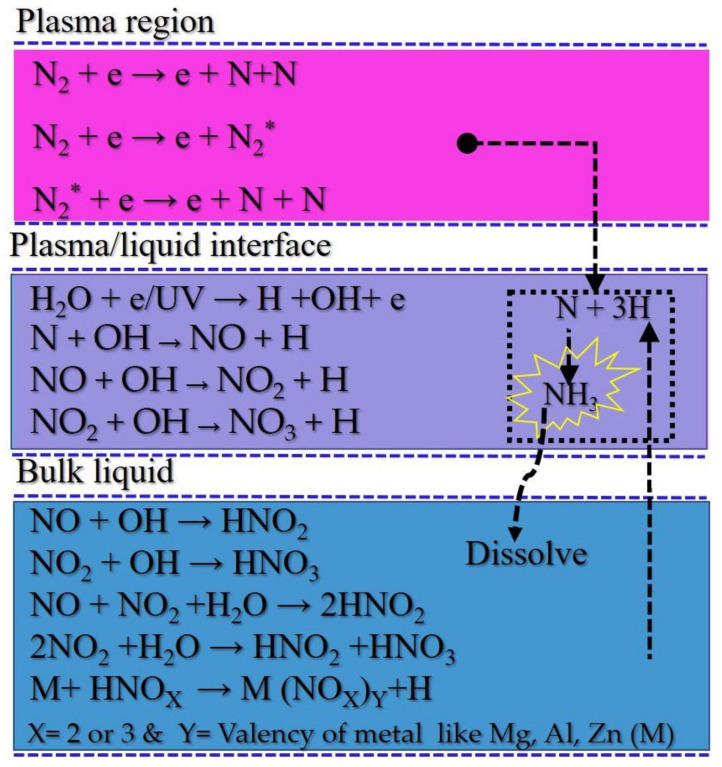
Chemical pathways of plasma-assisted nitrogen fixation and acid neutralization.

Plasma-assisted nitrogen fixation reaction pathways and acid neutralization as explained in Equations (1)–(22) is also summarized in Figure 10. The neutralization of plasma-generated acids with the help of metals is possible only if the standard reduction potential (E${}^{o}$) is negative. Neutralization and generation of nascent hydrogen is promoted as the reduction potential becomes more negative [7,64]. In this experiment, Mg, Al, and Zn were selected, and their standard reduction potentials are −2.37, −1.67, and −0.76, respectively; thus, ammonia synthesis rates and pH values for the different activated waters increase in the order Zn-PAW < Al-PAW < Mg-PAW, as listed in Table 1. Metal whose E${}^{o}$ is positive cannot generate nascent hydrogen from the plasma-treated water, thus the metal like copper is not much suitable for neutralization [7]. Air as a working gas leads closer to the real applications of this technology. However, air plasma is not appropriate for reduction of N into NH${}_{3}$, which is already explained in our previous report [9]. The nitrogen oxidation rate is faster than reduction rate. Thus, air working gas is favorable to NO_X_ generation and harsher to NH${}_{3}$ synthesis.

**Figure 11:**
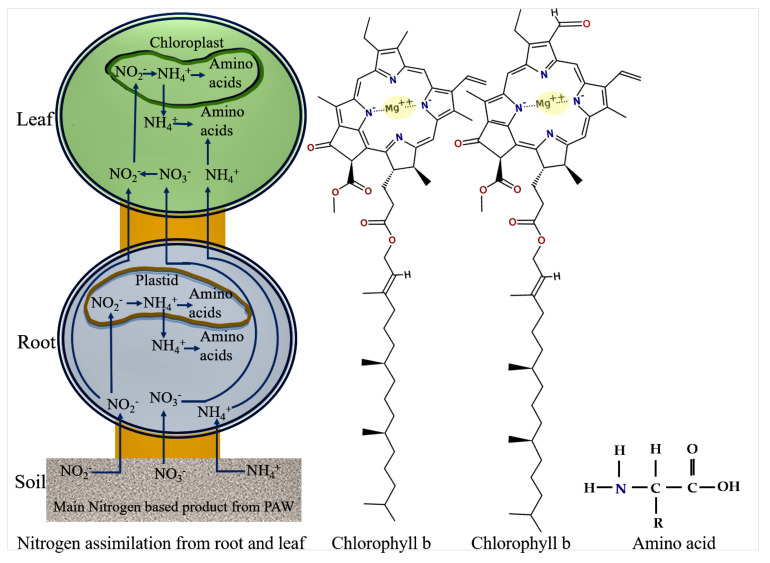
Schematics showing general nitrogen assimilation in the roots and leaves of plants and the chemical structures of chlorophyll and amino acids.

Biomolecules are mainly formed from carbon, water, nitrogen, and minerals. Plants can directly absorb carbon from carbon dioxide during photosynthesis, water from the ground, and minerals and nitrogen-based compounds from fertilizer. For plant uptake, the basic accessible nitrogen derivatives are NH${}_{4}^{+}$, NO${}_{2}^{-}$, and NO${}_{3}^{-}$ [11,68]. NO_X_ in water has stable anions which are involved in signaling during the biophysiological processes of the plant. For example, NO${}_{2}^{-}$ is advantageous for inducing seed germination, as it is involved in the regulation of dormancy-linked endogenous hormones of seeds [19,20,69]. An abundance of nitrogen in plants can be observed as a deep green color of the leaves, increased protein content, and increased grain plumpness [2]. In general, higher nitrogen availability lead to increases in plant productivity [53]. Plants are made up of a series of nitrogen-sensitive membranes. These respond and absorb fixed nitrogen derivatives from the soil, in the so called “nitrogen responses” of the plant [70]. Nitrogen responses are involved in morphological and physiological changes in plants that can produce higher crop yields [71]. Fixed nitrogen and metal ions are absorbed by the rhizosphere, allocated to root transporters and then assimilated by the various part of the plant [70]. The nitrogenous compounds found in PAW w/wo metals are at least in part responsible for the increases in plant growth and dry weights that we observed. The general mechanism of nitrogen assimilation is presented in Figure 11 along with the chemical structures of chlorophyll and the amino acid (building blocks of proteins).

In limited concentrations, metal ions such as magnesium (Mg${}^{++}$), aluminum (Al${}^{+++}$), and zinc (Zn${}^{++}$) play beneficial roles in plant physiology [55]. The maximum limit of these cations varied by the nature of the plant. For example, the optimum level of Zn${}^{++}$ for wheat crop is 3 mM, whereas for the tea plant, it is only 30 M [55]. Overall, the suggested quantity of nitrogen per 10,000 m${}^{2}$ is 200 kg [72]. Limits for the nitrogen content, metal ion contents, and pH value can vary with the treatment time and volume of the target liquid. Adverse effects on the germination rate and growth and development of plant had been observed for long-term plasma treatment, because of oxidative stress generated by the RONS [73]. Overdoses of the metal ions are also toxic for plants [55]. These ions also influence the roles of various enzymes and proteins. Advancements in germination and seedling growth may be the result of limited doses of these dissolved metal ions in the plant system, as explained in elsewhere [55]. Metal ions can enhance the cellular metabolism and support the roles of enzymes in boosting protein and chlorophyll syntheses [55].

Chlorophyll is crucial for photosynthesis. It assists plants to obtain energy from light [74]. Assimilated nitrogen is used to synthesize chlorophyll. Chlorophyll absorbs mostly the blue and red components of the visible range of the electromagnetic spectrum, reflecting the light wavelengths of ∼600 nm, which results in its intense green tone [74]. It traps light energy from the sun, which is then used to combine carbon dioxide and water to produce sugars during photosynthesis [74]. Chlorophyll molecules are specifically arranged in protein complexes, which are embedded in the thylakoid membranes of chloroplasts [74]. There are two types of chlorophyll: chlorophyll a and b. In principal, chlorophyll a and chlorophyll b have identical roles, but chlorophyll b is more soluble in water than chlorophyll a, owing to its carbonyl group [74]. Mg is also a fundamental constituent of chlorophyll and is involved in the photosynthesis process. Thus, in Figure 8a, the Mg-PAW is seen to promote chlorophyll synthesis in plant leaves, to an extent greater than that seen for the other PAWs. Increases in chlorophyll optimize carbon assimilation in plants via photosynthesis. Pernollet et al. [75] discussed the relationship between photosynthesis and protein synthesis in corn plants. According to their report, the amino acids are mainly formed by photosynthetic metabolism of sucrose and glucose. Thus, the trend of total protein content in fresh plants among the different treatments, as shown in Figure 8b, follows that of the chlorophyll concentration, as shown in Figure 8a. Ultimately, increases in proteins within plants facilitate increases in crop yields.

In previous studies, plasma-treated water has been used to enhance seed germination and plant growth [1,2,71,76,77]. Most of the researchers realized that the pH of the plasma-treated water must be neutral for sustainable plasma agriculture because irrigation using acidic water will have negative effects, such as root burn, on plants. Long-term utilization of such acidic water also renders soil completely infertile. During the process of nitrogen oxidation, the pH of the plasma-treated water decreased down to as low as 2 [2,32,41]. Accordingly, it is necessary to neutralize plasma-treated water before any real field applications are attempted. The generation of nascent hydrogen by metals in the plasma-treated water neutralizes the plasma-treated water, increases the NH${}_{3}$ synthesis rate, and enhances the mineral content of the water. We performed several experiments involving various treatment times to identify an optimized plasma treatment for this sample volume, to promote the germination, growth, and development of plant (data not shown). A clear antagonistic effect on seed germination and plant growth was seen after irrigation for more than 15 min of plasma treatment in all trials, which may be due to an excessively large amount of metal ions and RONS in the irrigated water. Thus, the presented report consists of the results of water that underwent only 10 min of plasma treatment in the presence of various metals. Up to this plasma treatment time and volume, the water obtained can be directly used for irrigation without additional processing. The obtained neutralized, metal ion-rich, and nitrogen-fixed water can be directly used for advanced plasma agriculture. Plasma agriculture technique could be a milestone for both hydroponics and soil-based plasma agriculture [78]. It could also be adopted as a means of helping to maintain soil pH, as in this respect its effect contrasts with that of other inorganic fertilizers. The main progressive stages of crop development and their requirements in different growth environments, from hydroponics to soil substrates, should be discussed in future. However, this study provides a framework for the synthesis of plasma-based fertilizers by addressing the requirements of crops and their ecosystems.

## 4. Materials and Methods

### 4.1. Experimental Setup

**Figure 12:**
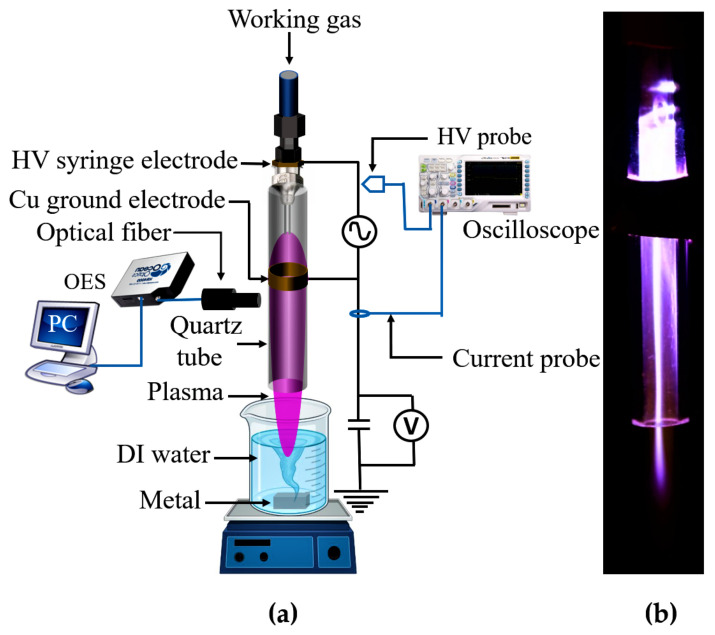
(**a**) Schematic of the atmospheric-pressure nitrogen plasma jet. (**b**) Photograph of the plasma source.

The design of the experimental system is shown in Figure 12a. A nitrogen plasma source is made by implanting a 50 mm long stainless steel needle into a 72 mm long quartz tube with external and internal diameters of 4 and 2 mm, respectively. A 5 mm wide copper tape electrode was wrapped around the quartz tube 5 mm below the lower tip of the needle. When an AC source (10 kV, 33 kHz) was applied to the needle together with 1000 sccm of N${}_{2}$ gas, plasma was produced and then propagated along with the gas. The plasma was directly exposed to 40 mL of deionized (DI) water. The distance between the surface of the water and the nozzle of the quartz tube was maintained at 3 mm. Furthermore, a 5 g piece of Zn, Mg, or Al was immersed in the DI water. An image of the plasma jet is also displayed in Figure 12b.

### 4.2. Electrical and Optical Properties of Plasma Jet

Discharge voltages and current waveforms were measured using a high-voltage probe (Tektronix P6015A) and current probe (LeCroy CP030), respectively. The measured voltages and currents were recorded using a wide-band digital oscilloscope (LeCroy wave surfer 434 MHz). A 33 nF capacitor was connected in series with the grounded electrode. The voltage across this capacitor was recorded to estimate the transferred charges. The Lissajous loop was constructed by plotting the charges (Q) versus the applied voltage (V). The Q–V Lissajous figure is a closed loop. The area enclosed by this loop was calculated by integrating the Q–V Lissajous figure which is equal to the energy dissipated by plasma in each cycle as given by Equation (23) [45,46,48]:

(23) $$\begin{array}{c} E=\int_{t=0}^{t=T}\mathrm{VIdt}=A_{\mathrm{lissajous}}. \end{array}$$

Before any real-life applications of a source can be realized, it is necessary to estimate the energy consumption rate. Energy yield in g of ammonia synthesis per kWh (=3.6 MJ) was calculated by using following relation (24) [40]:

(24) $$\begin{array}{c} \mathrm{Energy}\;\mathrm{yields}\;=\frac{\mathrm{Concentration}\;\mathrm{in}\;g/L\times \mathrm{Volume}\;\mathrm{in}\;L}{\mathrm{Power}\;\mathrm{in}\;\mathrm{kW}\times \;\mathrm{Time}\;\mathrm{in}\;h\;}. \end{array}$$

During plasma discharge, numerous RONS are generated and delivered to the target liquid. OES is one of the best and easiest methods for monitoring the generation of these species in the gas phase [54]. Similarly, OES was carried out on the plasma using a spectrometer (HR4000+CG-UV-NIR, Ocean Optics, Inc., Orlando, FL, USA) and a 400 mm diameter optical fiber. The spectrometer was wavelength calibrated using an Hg-Ar lamp (model 6048, Newport Corporation, Irvine, CA, USA). Integration time of OES was 600 s and resolution of the spectrometer is 0.27 nm.

### 4.3. Evaluation of Physical and Chemical Properties of Plasma-Treated Water

The chemical content of the plasma-treated water (the NO_X_, H${}_{2}$O${}_{2}$, and NH${}_{3}$ contents in water was assessed using assay kits for which all the reagents were purchased from Bio-Assay Systems. Detailed methodologies for the measurement of these chemical species have already been reported in our previous manuscripts [9,53,53]. For quantitative evaluation of the total NO, NO${}_{2}^{-}$, and NO${}_{3}^{-}$ contents, an improved Griess reagent kit was used. Concentrations were measured with the help of a standard calibration curve obtained from a plot of known NOx concentrations versus absorbance at 540 nm [13]. The NH${}_{3}$ concentrations were measured using an improved o-phthalaldehyde method [13,79]. A standard NH${}_{3}$ calibration curve obtained from known concentrations versus the fluorescence emission intensity at 450 nm. Fluorescence was obtained by excitation at 360 nm, and the concentration of NH${}_{3}$ in the sample was estimated from the fluorescence intensity. Similarly, a quantichrome peroxide assay kit was used to measure the amount of H${}_{2}$O${}_{2}$ present in the sample from the 585 nm absorbance produced by mixing the reagent with H${}_{2}$O${}_{2}$ [53] The fluorescence intensity and absorbance were measured using a BioTeK Gen 5 micro-plate reader. Similarly, NO in gas phase was measured by portable tetrahydrothiophene nitric oxide detector.

### 4.4. Measurement of Germination Rates and Plant Growth

To determine the effects of PAW w/wo metals on seed germination and plant development, trials of 50 seeds were placed in 5 Petri dishes (each contained 10 seeds). The seeds were covered by a layer of cotton. The seed samples were watered with 5 mL of deionized (DI) water, PAW, PAW with Zn (Zn-PAW), PAW with Al (Al-PAW), or PAW with Mg (Mg-PAW) daily and maintained in a dark chamber at 25 ± 2 ${}^{\circ }$C and 75 ± 5 relative humidity. The number of germinated seeds was recorded each day, and the germination rate was calculated from Equation (25) [2]:

(25) $$\begin{array}{c} \mathrm{Germination}\;\mathrm{rate}=\frac{\mathrm{Number}\;\mathrm{of}\;\mathrm{germinated}\;\mathrm{Seed}}{\mathrm{Total}\;\mathrm{number}\;\mathrm{of}\;\mathrm{seeds}}\times 100\%. \end{array}$$

The germination rate was assessed over days 2–5 after sowing. Another 15 seeds for each set of trials was sowed in separate nursery pot each of them contained 50 g of vermiculite. Every 48 h, 10 mL of water was used to irrigate the vermiculite. The plants were cultivated in an isolated cabinet whose temperature was controlled at 25 ± 2 ${}^{\circ }$C, humidity was maintained at 75 ± 5%, and a 16 h light/8 h dark cycle was used. At the end of the 6th day after sowing, the shoot lengths of the corn plants, including the leaves, were measured, and this measurement was repeated daily until 10 days after sowing. Then, the plant was gently removed from the vermiculite and the various physiological changes that had occurred to the plant were studied.

### 4.5. Determination of Chlorophyll Content of Fresh Leaves

Seedling leaf samples from different treatments were collected on the 10th day after sowing. Each sample consisted of 2–3 leaves. The fresh leaves were cut into small pieces and thoroughly mixed. A sample of 0.2 g was weighed and transferred to a 25 mL test tube, to which 80% acetone was added to volume. The test tube was inverted several times to ensure that all the leaves were washed with the acetone solution. The test tube then was covered with aluminum foil to block daylight, before being incubated at room temperature for 3–4 days until the leaves had turned completely white. The extracted liquid was filtered, and the absorbance of 200 µL of the liquid placed in a clear-bottom 96-well plate was measured at wavelengths of 663 and 645 mm, with the help of a plate reader (Synergy HTX Multi-Mode Reader from Bio-TeK Instruments, Winooski, VT, USA). The amounts of chlorophyll a and chlorophyll b and the total chlorophyll were calculated from Equations (26)–(28) [74].

(26) $$\begin{array}{c} \mathrm{Chlorophyll}\;a=(12.72\times A_{663}-2.69\times A_{645})\times X/1000n. \end{array}$$

(27) $$\begin{array}{c} \mathrm{Chlorophyll}\;b=(22.88\times A_{645}-4.68\times A_{663})\times X/1000n. \end{array}$$

(28) $$\begin{array}{c} \mathrm{Total}\;\mathrm{Chlorophyll}=(8.02\times A_{663}-20.34\times A_{645})\times X/1000n. \end{array}$$

where $A_{663}$ = absorption intensity at 663 nm, $A_{645}$ = absorption intensity at 645 nm, X = total filtrate volume, and n = tissue weight. The results were obtained in units of mg per g of fresh weight.

### 4.6. Determination of Total Soluble Protein Content of Fresh Leaves and Roots

For protein determination, 0.1 g of fresh leaf and root samples was frozen using liquid nitrogen. The resultant ground dust was mixed with 1 mL of phosphate-buffered saline (PBS) and then centrifuged at 20,000 *g* for 15 min. This solution was maintained in an ice bath until total decantation. After decantation, the isolated solution was transferred to another test-tube. The total soluble protein content was assayed using the Bradford (Bio-Rad, Hercules, CA, USA) method with bovine serum albumin as a standard [80].

### 4.7. Determination of Dry Weight

After the 10th day after sowing, the seedlings were cleaned with water and divided into two parts: roots and shoot. Then, these separated roots and shoot samples were dehydrated at 65 ${}^{\circ }$C for 5 days in a dry oven until the sample weight stabilized. The dry weight of each sample was then measured.

### 4.8. Measurement of Ammonia (NH${}_{3}$) or Ammonium Ion NH${}_{4}^{+}$) in Plant Tissue

For NH${}_{3}$ or NH${}_{4}^{+}$ determination, 10 mg of dried leaf or root powder was extracted with 1 mL of DI water by incubated at room temperature for 15 min with shaking. After the incubation, this solution was then centrifuged for 5 min at 12,000 *g* and the supernatant was transferred into new tube. The concentration of the NH${}_{3}$ or NH${}_{4}^{+}$ present in the dry corn sample was measured by mixing 0.4 mL of water, 2.5 mL of phenol-sodium nitroprusside, and 2.5 mL of alkaline hypochlorite (125 mM NaOH, 5 ppm NaOCl) with 0.1 mL of the sample suspension in water. The resultant mixtures were incubated at 30 ${}^{\circ }$C for 30 min, after incubation the color had changed to blue. A 200 µL aliquot of this solution was then transferred to the clear flat-bottom 96-well plate, and the absorbance at 635 nm was acquired using same plate reader. (NH${}_{4}$)${}_{2}$SO${}_{4}$ was used as a standard and the total concentration of NH${}_{3}$ or NH${}_{4}^{+}$ was estimated from a standard calibrated curve plot of absorbance versus known concentration. This measurement is commonly known as the phenol-hypochlorite method [81].

### 4.9. Measurement of Nitrate Nitrogen (NO${}_{3}$-N) in Plants

A colorimetric technique was used to determine the total NO${}_{3}$-N concentration in dry corn plants. The technique was based on determination of the absorbance of nitrosalicylic acid in a basic solution. The nitrosalicylic acid was generated as a response of the plant tissue to treatment with salicylic acid in 95% concentrated H${}_{2}$SO${}_{4}$. Similar to the measurement of the ammonium ion, 0.1 mg of dry leaf and root samples were added to 1 mL of DI water. The mixture was incubated at 45 ${}^{\circ }$C for a hour and then filtered with filter paler. The standard reagents were prepared follows: (a) A 500 mg/L nitrate standard solution was prepared from potassium nitrate. Solutions with known reagent concentrations (10, 20, 30, 40, 50, 60, 80, and 100 mg/L) were prepared by dilution and maintained at 4 ${}^{\circ }$C. (b) Salicylic acid (5 g) was mixed into 100 mL of conc. sulfuric acid to prepare 5% (mass/volume) salicylic acid 5% (mass/volume) in 95% concentrated H${}_{2}$SO${}_{4}$. (c) 2N NaOH was prepared by dissolving 40 g of NaOH in DI water to make a 500 mL volume of solution.

Aliquots (0.1 mL) of standard samples and standard solutions were mixed with 0.4 mL of the salicylic acid solution in a 30 mL tube. After 20 min incubation at room temperature, 9.5 mL of the 2N NaOH solution was added to samples and standards. The NO${}_{3}$-N concentration was estimated from the absorbance of 388 and 440 nm using a plate reader. This protocol is thoroughly described in a previous report [82].

### 4.10. Statistical Analysis

For each sample, at least three repeated experiment were carried out and the average values are presented. Error bars indicate ±1 standard deviation. *T*-tests were used for statistical analysis. The symbol * designates for t-test result which rejects the null hypothesis at the 5% significance level.

## 5. Conclusions

In this experiment, hydrogen was generated by the reduction of hydrogen ions found in plasma-generated acid (HNO${}_{2}$ or HNO${}_{3}$) with the help of electrons produced by oxidized metals in water. This process not only enhanced plasma-assisted nitrogen fixation by providing additional hydrogen via a nitrogen reduction reaction, but also neutralized the plasma-treated water. The pH value and nitrogen fixation rate were found to directly depend on the negative reduction potential of an immersed metal. The effect of enhanced plasma-assisted nitrogen fixation in water containing an immersed metal on the germination rate and growth of corn plants (Zea mays) were investigated. The neutralized, metal ion containing plasma-treated water could be used directly on plants as a fertilizer. Compared to DI water, this plasma-activated water enhanced the seed germination rate and plant development. The utilization of neutralized, metal ion-rich, nitrogen-fixed water to irrigate plants resulted in positive physiochemical changes in the plants. The plants responded to the accessibility of nitrogen species such as NH${}_{4}^{+}$, NO${}_{2}^{-}$, and NO${}_{3}^{-}$ by producing an intense green color in their leaves, in accordance with increases in the chlorophyll and protein content. Based on our experiments, much greater yield boosts could be achieved in short periods with the assistance of irrigation using metal ion neutralized nitrogen plasma-treated water.

## Figures and Tables

**Table 1:** Physicochemical properties of DI water, PAW, PAW with Zn (Zn-PAW), PAW with Al (Al-PAW), and PAW with Mg (Mg-PAW).

Activated Water	NH${}_{3}$ (mg/L)	NO_X_ (M)	H${}_{2}$O${}_{2}$ (M)	pH
DI water (control)	0.0	0.0	0.0	6.8 ± 0.2
PAW	2.1 ± 0.1	490.0 ± 53.7	38.2 ± 5.0	4.3 ± 0.3
Zn-PAW	2.6 ± 0.3	520.7 ± 71.6	35.3 ± 3.1	4.7 ± 0.2
Al-PAW	2.9 ± 0.2	450.1 ± 69.5	32.6 ± 6.5	5.1 ± 0.3
Mg-PAW	4.9 ± 0.3	597.5 ± 53.4	28.7 ± 4.2	6.2 ± 0.4

## Data Availability

The data that support the findings of this study are available from the corresponding author upon reasonable request.
